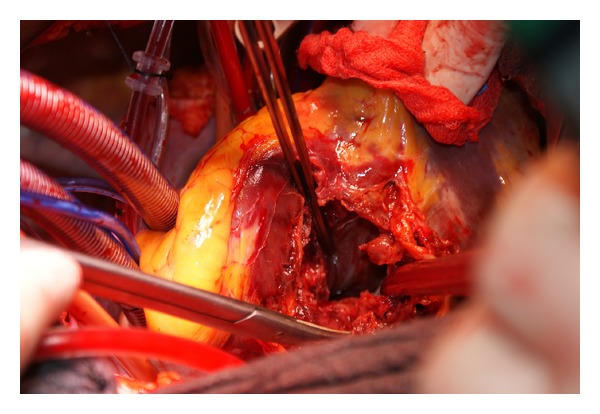# Fatal Huge Left Free Wall Ventricular Rupture after Acute Posterior Myocardial Infarction

**DOI:** 10.1155/2013/691971

**Published:** 2013-10-08

**Authors:** Francesco Formica, Silvia Mariani, Orazio Ferro, Giovanni Paolini

**Affiliations:** ^1^Cardiac Surgery Unit, San Gerardo Hospital, Monza, Italy; ^2^Department of Surgery and Interdisciplinary Medicine, University of Milan-Bicocca, Italy; ^3^U.O. Cardiochirurgia, A.O. San Gerardo Via Pergolesi 33, 20900 Monza (MB), Italy

## Abstract

A 77-year-old man, with a recent history of an acute inferior myocardial infarction, was referred to our hospital with echocardiographic and clinical signs of left ventricular free wall rupture (LVFWR). The intraoperative finding demonstrated a huge double LVFWR. The inferoposterior wall was dramatically destroyed without any possibility to repair.

Cardiac rupture represents a catastrophic complication of myocardial infarction with an incidence of 6% in the prereperfusion era [1]. In the reperfusion era, its incidence is between 1% and 3% of all myocardial infarction patients [2]. Despite significant improvement in the diagnosis and therapy of myocardial infarction, in-hospital death in patients complicated by cardiac rupture remains dramatically high. 

We describe the case of 77-year-old man who was admitted to peripheral hospital with chest pain and mild ST elevation on D2, D3, and aVF leads at the time of electrocardiogram admission. Diagnosis of acute posterior-inferior myocardial infarction was made, and the patient underwent prompt cardiac catheterization, which showed a proximally total occlusion of the right coronary artery. Due to initial symptoms of low cardiac output, a transthoracic echocardiogram was performed and pericardial effusion was detected. Therefore, the patient was referred to our hospital with echocardiographic and clinical signs of pericardial tamponade with the suspicion of left ventricular free wall rupture (LVFWR) to undergo emergently surgical repair. The patient arrived to our unit about 2 hours after initial symptoms. On arrival to operating room, the patient showed clinical signs of low cardiac output despite conventional therapy with inotropes and vasoconstrictor; the blood pressure was 80/50 mmHg, the pulse rate was 65 beats/min, the extremities were cold, and the urine output was less than 0.5 mL/Kg/min. The patient was promptly intubated and ventilated. A standard longitudinal sternotomy was performed, and the pericardium was opened. A fresh clot was observed over the inferior left ventricular wall. The systolic pressure dramatically raised, but suddenly a huge bleeding was observed into the pericardial cavity, and a pulseless ventricular tachycardia occurred. A sinus rhythm was obtained after internal DC shock at 7 Joule, cardiopulmonary bypass was established immediately, and the heart was arrested. The intraoperative finding showed a huge double LVFWR. One rupture was located in the territory of posterior descending artery for a length of about 6 cm (Figure 1, ∗ mark), while the other rupture was located along the course of coronary sinus for a length of about 7 cm (Figure 1, # mark). The inferoposterior wall of the left ventricle was hugely destroyed (Figure 2) without any possibility to be repaired. 

Risk factors related to cardiac rupture that have been identified to date include anterior location of the infarct with ST-segment elevation, ST deviation, positive initial cardiac biomarkers, age older than 70 years, female sex, and no history of previous angina or myocardial infarction. Mortality remains high as reported by the Global Registry of Acute Coronary Events (GRACE) registry [1]. In this registry, hospital mortality rate was 80% in LVFWR patients versus 4.5% in those without cardiac rupture. The majority of patients die shortly after LVFWR from instantaneous pericardial tamponade and hemodynamic collapse. However, up to one-third of cases are subacute in nature, allowing limited time for emergent surgical repair to prevent sudden death.

When the cardiac rupture is limited to a small myocardial area and the rupture is not complete (subacute type), the probabilities to repair the left ventricle are higher than in patients with acute cardiac rupture type, and the surgical repair can be performed even without cardiopulmonary bypass with acceptable early and late results [3].

In our patient, the large amount of myocardial tissue destroyed made it impossible for any type of surgical technique of repair. Very extreme surgical options such as heart transplantation, insertion of left ventricular assist device [4], or total artificial heart can be seen, but they are certainly not part of a routine surgical strategy, because not all cardiac surgery centers can provide immediately these alternatives.

## Figures and Tables

**Figure 1:**
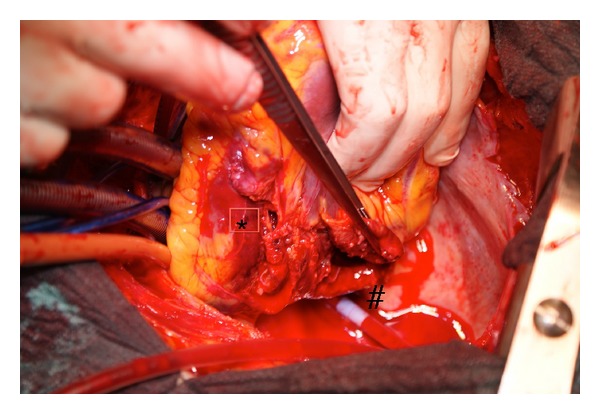


**Figure 2:**